# Pyroptosis-triggered pathogenesis: New insights on antiphospholipid syndrome

**DOI:** 10.3389/fimmu.2023.1155222

**Published:** 2023-03-31

**Authors:** Yuan Tan, Qi Liu, Zhongxin Li, Shuo Yang, Liyan Cui

**Affiliations:** ^1^ Department of Laboratory Medicine, Peking University Third Hospital, Beijing, China; ^2^ Core Unit of National Clinical Research Center for Laboratory Medicine, Peking University Third Hospital, Beijing, China; ^3^ Institute of Medical Technology, Peking University Health Science Center, Beijing, China

**Keywords:** APS, pyroptosis, mechanisms, cellular effects, therapy

## Abstract

APS (antiphospholipid syndrome) is a systematic autoimmune disease presenting with the high levels of aPLs (antiphospholipid antibodies). These autoantibodies are involved in various clinical manifestations, mainly including arterial or venous thrombosis formation, proinflammatory response, and recurrent pregnant loss. Pyroptosis is a form of lytic programmed cell death, and it aggravates autoimmune diseases progression *via* activating NOD-like receptors, especially the NLRP3 inflammasome and its downstream inflammatory factors IL (interleukin)-1β and IL-18. However, the underlying mechanisms of pyroptosis-induced APS progression remain to be elucidated. ECs (endothelial cells), monocytes, platelets, trophoblasts, and neutrophils are prominent participants in APS development. Of significance, pyroptosis of APS-related cells leads to the excessive release of proinflammatory and prothrombotic factors, which are the primary contributors to APOs (adverse pregnancy outcomes), thrombosis formation, and autoimmune dysfunction in APS. Furthermore, pyroptosis-associated medicines have made encouraging advancements in attenuating inflammation and thrombosis. Given the potential of pyroptosis in regulating APS development, this review would systematically expound the molecular mechanisms of pyroptosis, and elaborate the role of pyroptosis-mediated cellular effects in APS progression. Lastly, the prospective therapeutic approaches for APS would be proposed based on the regulation of pyroptosis.

## 1 Introduction

APS (antiphospholipid syndrome) is considered as a systemic autoimmune disease accompanied with inflammatory response, venous or arterial vascular thrombosis, recurrent fetal loss, and early pregnancy loss or pregnancy morbidity (1). Most of APS patients undergo multiple organs-systems injury due to the presence of at least one of serum aPLs (antiphospholipid antibodies), including aCL (anti-cardiolipin antibodies), anti-β2GPI (anti-beta2-glycoprotein I) or LAC (lupus anticoagulant) (1). At present, APS is mainly composed of PAPS (primary APS) and CAPS (catastrophic APS) (2). CAPS, as the variant of APS, is a rare, life-threatening disease with acute multisystem thrombosis and SIRS (systemic inflammatory response syndrome) (2). Besides, APS may occur in other systemic autoimmune diseases, particularly SLE (systemic lupus erythematosus) (3).

Pyroptosis is regarded as an essential innate immune defense against pathogens infection (4, 5). It has received growing attention due to its contributions to various diseases, mainly involving in gasdermins-mediated programmed death (6). Gasdermin superfamily is composed of gasdermin A/B/C/D/E (GSDMA/B/C/D/E) and DFNB59 (Pejvakin, PJVK) in human (7). Among these conserved proteins, GSDMD and GSDME are most extensively studied in pyroptosis (8, 9). Except DFNB59, other gasdermin members consist of the N-terminal PFD (pore-forming domain) and the C-terminal RD (repressor domain) (7). The interaction of PFD with RD maintains the oligomeric construct of gasdermins (10). Upon activated by endogenous and exogenous stimulus, the N-terminal PFD of gasdermins is separated from the C-terminal RD by caspases or granzymes, then the N-terminal PFD oligomerizes to form pores in the cell membrane, driving cell swelling and rupture, inflammatory molecules production, and pyroptotic cell death (11).

ECs (endothelial cells), neutrophils, monocytes, trophoblasts, and platelets are key players of APS progression. A great deal of evidence has showed that pyroptosis of these cells causes autoimmune disorders and accelerates diseases progression by triggering inflammasome activation and thrombosis formation, such as RA (rheumatoid arthritis) and SLE (12, 13). Nonetheless, the role of pyroptosis in promoting APS progression remains to be profoundly explored, which would reveal novel mechanisms for improving the treatment strategies of APS. Herein, this review would introduce the present molecular mechanisms of pyroptosis, and elucidate the contributions of pyroptosis of ECs, neutrophils, monocytes, trophoblasts, and platelets to APS development. In the last, the role of pyroptosis-related inhibitors in optimizing APS therapy would be exhibited.

## 2 Molecular mechanisms of pyroptosis

**Figure 1:**
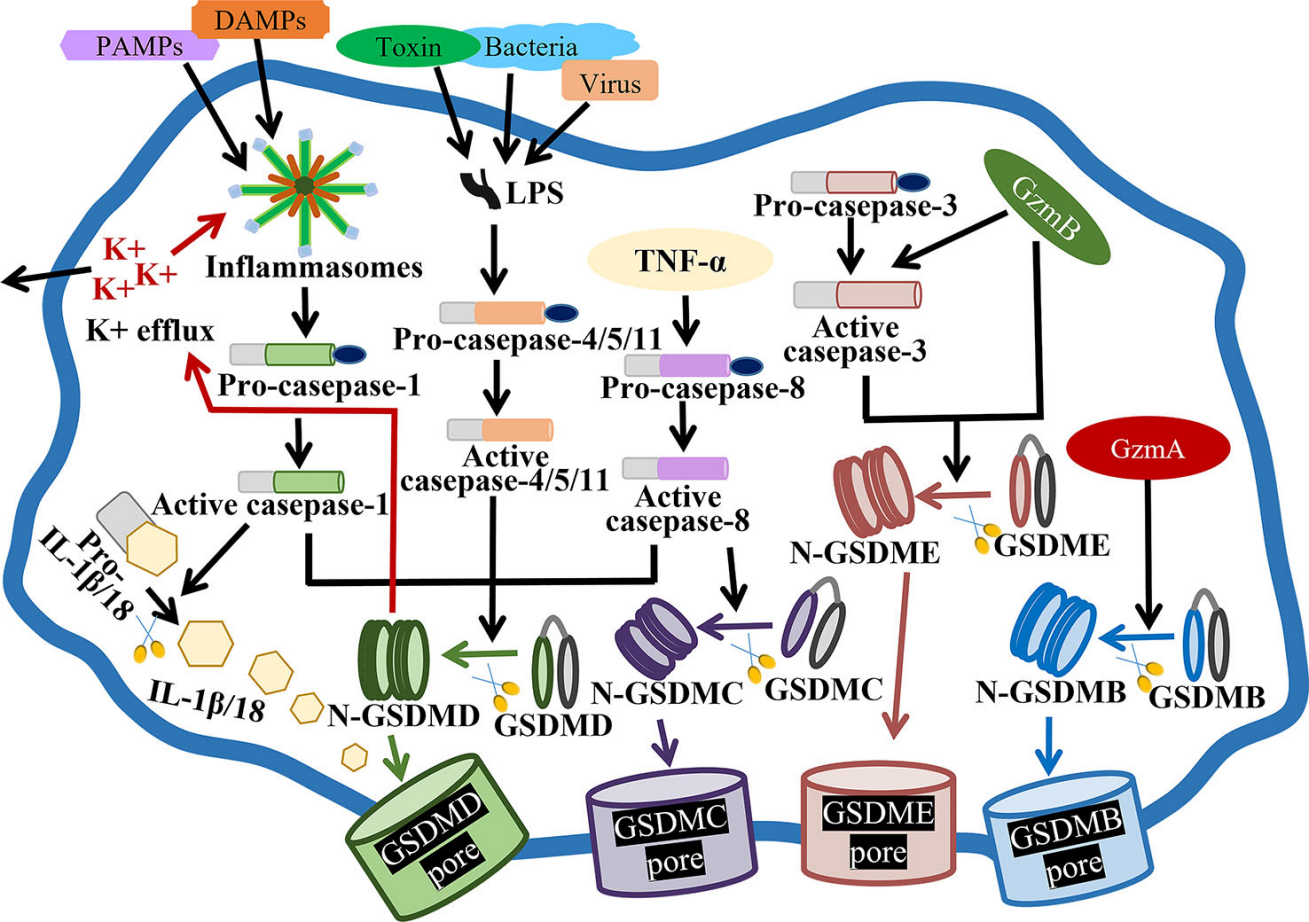
Molecular mechanisms of pyroptosis. In the canonical pathway, PAMPs and DAMPs receive intracellular signaling to stimulate inflammasomes and active caspase-1. Cleaved-caspase-1 cleaves GSDMD, pro-IL-1β and pro-IL-18. N-GSDMD perforates the cell membrane by forming nonselective pores, further causing cell lysis and death. In addition, IL-1β and IL-18 are secreted from the pores formed by N-GSDMD. In the noncanonical pathway, virus, bacteria or toxin-secreted LPS activates caspase-4, -5 and -11, triggering pyroptosis by cleaving GSDMD. In addition, the cleavage of GSDMD results in efflux of K+, ultimately mediating the assembly of NLRP3 inflammasome, resulting in the cleavage of pro-IL-1β and pro-IL-18. In the caspase-3-mediated pathway, active caspase-3 cleaves GSDME to form N-GSDME, inducing pyroptosis. In the caspase-8-mediated pathway, TNF-α treatment induces the activation of caspase-8, which cleaves GSDMD and GSDMC, resulting in pyroptosis. In the granzyme-mediated pathway, released GzmB rapidly activates caspase-3 and GSDME, causing extensive pyroptosis, and GzmB also directly activates GSDME. In addition, GzmA hydrolyzes GSDMB and directly activates GSDME.

As we all known, pyroptosis is mainly composed of the caspase‐1-dependent canonical pathway and the caspase‐4/5/11-mediated non‐canonical pathway (14) (**Figure 1**). Canonical pyroptotic cell death is regulated by inflammasomes, like NLRP3 (NLR family, pyrin domain containing 3), NLRP1, NLRC4 (NLR family CARD domain containing 4), AIM2 (absent in melanoma 2), and pyrin, consequently augmenting GSDMD and caspase-1 cleavage, as well as IL-1β and IL-18 release (15). The assembly of inflammasomes is initiated by PRRs (pattern recognition receptors), which are capable of recognizing PAMPs (pathogen-associated molecular patterns) and DAMPs (danger-associated molecular patterns) to boost downstream proinflammatory cytokines release (16). Caspase-4/5/11-mediated noncanonical pyroptosis lacks the upstream sensors, but it can be directly activated by the binding of intracellular LPS (lipopolysaccharide) with the N-terminal CARD (caspase activation and recruitment domain), subsequently generating GSDMD pores and releasing matured IL-1β and IL-18 (17).

Besides, apoptosis-related caspases, such as caspase-3 and -8, are thought to stimulate gasdermins-mediated pyroptosis (**Figure 1**). For example, caspase-3-mediated GSDME cleavage causes pyroptosis or secondary pyroptosis *via* generating a N-GSDME fragment and forming perforated membranes (18, 19). In mouse macrophages, caspase-8 functions as a regulator of pyroptosis, also contributing to GSDMD pores formation and pyroptotic cell death (20). As well, caspase-8 specifically lyses GSDMC to produce a N-GSDMC fragment, and generates pores on the cell membrane to induce pyroptosis (21).

Granzymes have been reported to kill target cells by inducing pyroptosis, such as GzmB (granzyme B) and GzmA (**Figure 1**). GzmB has been identified to activate caspase-3, then caspase-3 cleaves GSDME and induces GSDME-mediated extensive pyroptosis (22). Moreover, GzmB can directly cleave GSDME and cause pyroptosis (23). Additionally, lymphocytes-derived GzmA has been proved to cleave GSDMB at Lys229/Lys244 site, hydrolyze gasdermins at non-aspartic acid sites, form pores, stimulate pyroptosis and kill GSDMB-positive cells (24).

## 3 The role of pyroptosis in monocytes

Monocytes are key participants in natural immunity and host defense, engaging in aPLs-mediated inflammation, thrombosis, and autoimmune response (25). Monocytes dysfunction plays a prominent role in APS progression through producing inflammatory cytokines and TF (tissue factor) (26). Previously, pyroptosis has been reported as caspase-1-mediated monocytes death and is mainly detected in monocytes and macrophages. Hence, clarifying the underlying mechanisms of pyroptotic cell death of monocytes and macrophages is essential for alleviating APS development (**Table 1**).

**Table 1:** The pyroptosis-mediated pathological effects in APS progression.

Cells	Types	Mechanisms	Refs
Monocytes	Canonical	Phosphorylated ASC and LPC activate NLRP3 inflammasome and accelerate APS progression	(27, 28)
	Canonical	NLRC4 cooperates with NLRP3 to stimulate pyroptosis and aggravate inflammation	(29)
	Canonical	Infection induces AIM2 inflammasome-mediated pyroptosis by cleaving GSDMD	(30)
	Canonical	TLRs activation facilitates NLRP1 inflammasome assembly, caspase-1 activation and IL-1β secretion	(31)
	Canonical	Coordination of PTX3 with C1q enhances GSDMD-dependent pyroptosis by activating NLRP3	(32)
	Noncanonical	The binding of LPS with TLR4/MD-2 and caspase-4/11 boosts pyroptotic cell death	(33)
	Noncanonical	LPS/Ng strengthens TRAF3-induced ULK1 degradation, further stimulating IL-1β maturation and pyroptotic cell death	(34)
	Noncanonical	Overexpressed GSDMB promotes LDH release, triggering caspase-4/GSDMD-dependent pyroptosis	(35)
	Noncanonical	TNF heightens cell pyroptosis by activating caspase-3/GSDME pathway	(36)
	Noncanonical	Small molecule inhibitors of DPP8 and DPP9 induce caspase-1 and GSDMD-regulated pyroptosis	(37, 38)
Neutrophils	Canonical	Neutrophils undergo pyroptotic cell death *via* assembling NLRC4 and producing IL-1β	(39)
	Canonical	Infection induces neutrophils pyroptosis *via* ATP-mediated P2X7R signaling, resulting in NLRP3 activation, mature IL-1β and IL-18 release	(40, 41)
	Noncanonical	Caspase-8 cleaves GSDME to drive neutrophils pyroptosis	(42)
	Noncanonical	GSDMD is activated by LPS and cleaved by caspase-11, which plays a vital role in NETosis and TF release	(43)
ECs	Canonical	Acrolein, Cd and DBDPE promote ROS generation and NLRP3-mediated ECs pyroptosis	(44–46)
	Canonical	HMGB1 triggers proinflammatory response and ECs pyroptosis *via* activating RAGE/cathepsin B/NLRP3 signaling	(47)
	Canonical	HDAC11 promotes NLRP3/caspase-1/GSDMD-mediated vascular ECs pyroptosis	(48, 49)
	Noncanonical	RCN2 and SDHB enhance ROS generation, mitochondrial injury, and ECs pyroptosis	(50, 51)
	Noncanonical	Activated caspase-4 and -11 simultaneously stimulate GSDMD and GSDME, aggravating ECs pyroptosis	(52)
	Noncanonical	HDAC11 boosts caspase-3 and GSDME *via* interacting with ERG and decreasing ERG acetylation	(49)
	Noncanonical	LPS stimulates ECs pyroptosis by cleaving caspase-11 and GSDMD, resulting in mtDNA release	(53)
Trophoblasts	Canonical	Hyperactivated UPR boosts TXNIP/NLRP3 signaling to increase caspase-1, GSDMD, IL-18 and IL-1β production	(54)
	Canonical	HOXA9 boosts AMPK/TXNIP/NLRP3 inflammasome and pyroptosis by increasing chemerin transcription	(55)
	Canonical	The upregulated miR-124-3p triggers trophoblasts pyroptosis by boosting ROS/NLRP3/caspase-1/GSDMD axis	(56)
	Noncanonical	Overexpressed lncRNA NEAT1 heightens TLR4 transcription and LPS-induced trophoblasts pyroptosis	(57)
	Noncanonical	The downregulated LINC00240 aggravates oxidative stress-induced trophoblasts pyroptosis by increasing miR-155 and reducing Nrf2 expression	(58)
	Canonical	Activated TLR4/NF-*κ*B/PFKFB3 facilitates NLRP3-induced trophoblasts pyroptosis	(59)
Platelets	Canonical	S100A8/A9 induces platelets pyroptosis dependent on the activity of GSDMD and TLR4/ROS/NLRP3/caspase-1	(60)
	Canonical	HMGB1 boosts platelets pyroptosis *via* activating TLR4/ROS/NLRP3 and ASC	(61, 62)
	Canonical	ND stimulates platelets pyroptosis by producing mitochondrial superoxide and activating NLRP3	(63)

During the process of canonical pyroptosis, activated monocytes release caspase-1, GSDMD, IL-1β and IL-18 (64). Moreover, the plasma membrane damage of monocytes and macrophages causes K+ efflux and NLRP3 activation, subsequently triggering pyroptosis and accelerating APS progression (65). Mechanically, NLRP3 inflammasome in monocytes is activated by cAbl kinase-mediated ASC phosphorylation at Y146 site and LPC (lysophosphatidylcholine) (27, 28), further promoting IL-18 and IL-1β release. Particularly, Abl kinase inhibitors provide a novel therapeutical intervention for APS by reducing ASC phosphorylation, further blocking monocytes pyroptosis and inflammatory cytokines release (27). In addition, NSA (necrosulfonamide) can directly bind with GSDMD at Cys191 site and prohibit p30-GSDMD pores formation in both human monocytes and macrophages, further blocking NLRP3- and pyrin-mediated pyroptotic cell death and IL-1β release. Most importantly, NSA does not affect other innate immune pathways or other cell death pathways, suggesting a promising therapeutical strategy for ameliorating inflammatory reactions in APS (66).

NLRC4, another canonical inflammasome, is reported to cooperate with NLRP3 to stimulate inflammation in human macrophages, indicating an unexpected overlap between distinct inflammasome scaffolds (29). Besides, monocytes infection activates AIM2 inflammasome, caspase-1 and GSDMD cleavage, and the expression level of GSDMD on the cell membrane and cytoplasm of monocytes is linked with the severity of infection (30). Concerning NLRP1 inflammasome, it is prominently located in the nucleus of monocytic THP-1 cells and is closely correlated with the genetical risk of several autoimmune diseases (31). Also, the assembly of NLRP1 inflammasome is facilitated by TLRs (toll-like receptors) activation, subsequently inducing caspase-1 activation and bioactive IL-1β secretion (31). Of significance, treatment of monocytes with SB (silibinin) downregulates NF-κB pathway and inactivates NLRP1/NLRP3 inflammasomes in pregnant women with PE (preeclampsia), suggesting a prospective therapeutical strategy for APS-related APOs *via* abrogating inflammation (67). In these pyroptotic monocytes and macrophages, canonical inflammasomes would trigger thrombosis and accelerate APS progression *via* promoting TF release. And the deficiency of caspase-1 or GSDMD is identified to block flow restriction–induced venous thrombosis (68), but also renders monocytes and macrophages insensitivity to pyroptotic cell death (69). These findings reveal that targeting canonical pyroptosis of monocytes might improve APS therapy.

Endotoxic LPS is an essential stimulator of noncanonical pyroptosis pathway. TLR4/MD-2 (myeloid differentiation-2) complex and caspase-4/11 are extracellular and intracellular LPS receptors, respectively. Upon stimulated by LPS, TLR4/MD-2 complex is highly expressed on the surface of macrophages, monocytes, and ECs, then directly interacting with the lipid A motif of LPS. Similarly, caspase-4/11 boosts monocytes pyroptosis by binding with diverse LPS variants (33). Additionally, the administration of LPS/Ng (LPS plus nigericin) strengthens TRAF3 (tumor necrosis factor receptor-associated factor 3)-modulated ULK1 (unc-51 like autophagy activating kinase 1) ubiquitin and degradation in THP-1 cells. Further, the downregulated ULK1 significantly induces AIF nuclear relocation and stimulates caspase-1 activation, consequently promoting IL-1β maturation and pyroptotic cell death (34). Presently, GSDMB has been confirmed to participate in autoimmune diseases. The highly expressed GSDMB not only promotes N-GSDMD cleavage and LDH (lactate dehydrogenase) release in monocytes, but also triggers caspase-4/GSDMD-dependent non-canonical pyroptosis by directly binding to the CARD domain of caspase-4. Therefore, GSDMB-mediated pyroptosis reveals a potential approach for relieving systemic inflammation in APS (35).

In particular, increasing clinical observations have detected the high levels of GSDMD or GSDME in several autoimmune diseases, and it is positively related with the disease activity. For instance, monocytes from RA patients are more susceptible to pyroptosis due to the higher GSDME level (36). Furthermore, TNF (tumor necrosis factor) treatment heightens pyroptosis of monocytes and macrophages by activating caspase-3/GSDME pathway. Caspase-3 inhibitor or silencing of GSDME dramatically inhibits TNF‐induced pyroptosis and alleviates arthritis, which might become a promising therapeutic method for APS-related inflammation (36). Likewise, another clinical evidence has shown that serum from RA patients promotes GSDMD-dependent pyroptosis of monocytes. Moreover, the coordination of PTX3 (pentaxin 3) with C1q remarkably enhances GSDMD-dependent pyroptosis by activating NLRP3 inflammasome, GSDMD and caspase-1 cleavage, and IL-6 release. Conversely, IL-6 strengthens PTX3 plus C1q-induced pyroptosis of monocytes, providing new insights for blocking pyroptosis-mediated persistent proinflammatory response in APS (32). In SLE patients, overexpressed GSDMD exerts a crucial function in the pathogenesis of monocytes and macrophages, and DSF (disulfiram) prominently represses GSDMD-mediated pyroptosis of THP-1 cells and relieves the disease severity (13).

Pyroptosis is a complicated formation of cell death, it is often accompanied by other biological processes, like apoptosis and necroptosis. Small molecule inhibitors of the serine peptidases DPP8 and DPP9 enhance pro-caspase-1 activation and GSDMD-regulated pyroptosis of monocytes and macrophages (37, 38). Meanwhile, caspase-1 induces apoptotic cell death in the absence of GSDMD *via* activating caspases-3 and -7 in monocytes. Further, caspases-3 and -7 cleave GSDMD at position D87 and hinder GSDMD-modulated pyroptosis, which shows a bidirectional crosstalk between pyroptosis and apoptosis pathways in monocytes and macrophages (37). On the other side, RIPK3 (receptor-interacting serine/threonine protein kinase 3)-mediated necroptosis and GSDMD-mediated pyroptosis collaborate to amplify inflammatory response in macrophages and ECs. Ablation of Ripk3 or Gsdmd efficiently prevents IL-1β maturation and release, revealing two potential targets for combined therapeutic interventions of APS (70).

## 4 The role of pyroptosis in neutrophils

Neutrophils are important leukocytes for the innate immune response, and they promote pyroptosis-mediated inflammation and thrombosis in APS patients (71). At the same time, pyroptosis of neutrophils stimulates neutrophils-mediated NETs (neutrophil extracellular traps) release and NETosis (concomitant cell death) (72). Further, NETs and NETosis have the capacity to affect autoimmunity, drive inflammatory cytokines production, as well as propelling thrombus formation by activating platelets and coagulation (73–75). Herein, pyroptosis-mediated signaling in neutrophils might execute a crucial function in APS pathology due to the pivotal roles of NETs and NETosis in thrombosis and inflammation (73–75) (**Table 1**).

In general, pyroptosis is initiated by intracellular pathogens infection, but the function of inflammasomes is different even between the two related immune cell lineages. For instance, canonical inflammasomes activation triggers caspase‐1-mediated macrophages pyroptosis, while they selectively activate caspase‐1/IL‐1β signaling without concomitant pyroptotic cell death in neutrophils (76, 77). Thereby, neutrophils might be resistant to pyroptosis and exhibit weaker GSDMD cleavage. In NLRP3 inflammasome-activated neutrophils, N-GSDMD is trafficked to azurophilic granules and neutrophils elastase is released into the cytosol, resulting in the secondary cleavage of GSDMD and mature IL-1β secretion, instead of accumulating N-GSDMD pores in the plasma membrane (78). Evidence has reported the resistant mechanisms of neutrophils pyroptosis (79). On the one side, the preserved mitochondrial membrane under active NLRP3 inflammasome leads to the pyroptosis resistance of neutrophils *via* mitochondria-dependent NLRP3 desensitization at DAMP-rich inflammatory region (77). The absence of neutrophils pyroptosis prolongs neutrophils’ lifespan, IL‐1β and IL‐18 production, and enables the clearance of microbial insult or cellular debris (76). On the other side, neutrophils might suppress SARM1 expression to subvert caspase‐1‐dependent pyroptosis, and the overexpressed SARM1 may stimulate caspase‐1‐dependent pyroptosis of neutrophils (80). Thereby, it would be relatively interesting to explore the contributions of neutrophils to inflammatory reactions in APS progression.

Despite the absence of GSDMD pores and the resistance of pyroptosis in neutrophils, several observations have detected pyroptotic cell death in neutrophils. For example, neutrophils can undergo pyroptotic cell death *via* assembling NLRC4 inflammasome and producing IL-1β (39). Another report suggests that infection induces neutrophils pyroptosis *via* ATP-mediated P2X7R signaling, resulting in cytoplasmic low K+ activation of NLRP3 inflammasome together with mature IL-1β and IL-18 release (40, 41). Furthermore, there is an interplay between apoptosis and pyroptosis. A study has found that RIPK1 can activate GSDME during bacterial infection. Then GSDME is cleaved by caspase-8, which does not only stimulate neutrophils lysis through activating extrinsic and intrinsic apoptosis, but also drive neutrophils pyroptosis (42).

Most significantly, pyroptosis may coordinate with NETosis to deteriorate the progression of autoimmune diseases (43, 81). It has been proved that NETosis can induce monocytes and macrophages pyroptosis to aggravate the secretion of inflammatory and prothrombotic factors, which might lead to plaque erosion and extensive thrombosis in APS patients (82). GSDMD, a pyroptosis executor, has been reported to facilitate NETs excretion from neutrophils. In noncanonical signaling, GSDMD is activated by intracellular LPS and cleaved by caspase-11, which plays a vital role in NETosis and TF release (43). For one thing, caspase-11 and GSDMD accelerate neutrophils plasma membrane rupture at the final stage of NETs release. For another thing, caspase-11 and GSDMD promote nuclear demodulation and DNA expansion at the early stage of NETosis (43). Fortunately, a pyrazolo-oxazepine scaffold–based molecule has been investigated to bind with GSDMD, further inhibiting NETosis (43). Accordingly, GSDMD inhibitors can be regarded as attractive therapeutical targets for forfending NETosis in APS.

## 5 The role of pyroptosis in ECs

Generally, pyroptosis of vascular ECs enhances vascular permeability and causes endothelial damage. This process would recruit more immune cells to adhere to the vascular wall, producing proinflammatory factors and promoting the formation of thrombotic plaques (83, 84). Current observations have demonstrated the key role of ECs pyroptosis in APS pathophysiology (**Table 1**).

ROS (reactive oxygen species) acts as the hub role in activating NLRP3 inflammasome and caspase-1-dependent canonical pyroptosis of ECs, and some environmental factors markedly stimulate ROS production. For example, treatment of human ECs with nicotine results in ROS/NLRP3/ASC activation, caspase-1/GSDMD-dependent pyroptosis and vascular endothelial injury (85). Acrolein, a common environmental pollutant, is linked with cardiovascular diseases, and exposure of HUVECs (human umbilical vein endothelial cells) to acrolein stimulates NLRP3 inflammasome assembly and pyroptosis *via* promoting ROS secretion in HUVECs (44). Cd (cadmium) is a crucial environmental pollutant associated with cardiovascular diseases, Cd-treated HUVECs increase ROS secretion and activate NLRP3 inflammasome, further strengthening pyroptosis of HUVECs (45). DBDPE (decabromodiphenyl ethane) is a novel environmental pollutant, it has been investigated to induce vascular endothelial injury and cardiovascular damage. DBDPE not only promotes ROS generation, but also causes NLRP3-mediated vascular ECs pyroptosis, as evidenced by the elevated NLRP3, ASC, and caspase-1 (46). As well, RCN2 (reticulocalbin-2) and SDHB (succinate dehydrogenase complex subunit B) are important regulators of ROS generation, mitochondrial injury, and ECs pyroptosis. And the inhibition of RCN2 or SDHB significantly attenuates pyroptosis, downregulates ROS and pyroptosis-related proteins (50, 51).

HMGB1 (high mobility group box 1) is a highly conserved and damage-related nuclear protein, the released HMGB1 can trigger proinflammatory response and canonical pyroptosis of ECs *via* activating RAGE (TLR4/advanced glycation end product)/cathepsin B/NLRP3 signaling (47). HDAC11 (histone deacetylase 11) is a class IV histone deacetylase, it promotes NLRP3/caspase-1/GSDMD-mediated vascular ECs pyroptosis under TNF-α treatment, and the HDAC11 inhibitor might prevent pyroptosis (48, 49). These canonical pyroptosis processes are characterized by the elevated caspase-1 cleavage, GSDMD expression, IL-1β and IL-18 production, LDH activity, and PI (propidium iodide) positive cells. And caspase-1 inhibitors can effectively repress pyroptosis, showing useful approaches for alleviating ECs damage-induced thrombosis in APS (85).

On the other side, the critical role of caspase-4/11 in modulating noncanonical ECs pyroptosis has been identified. In TNF-α-treated arterial ECs, activated caspase-4 and -11 simultaneously stimulate GSDMD cleavage and GSDME activity by binding with caspase-3. Both of the two pathways aggravate ECs pyroptosis and endothelial dysfunction, suggesting that caspase-4 and -11 are potential therapeutic targets of APS (52). Also, LPS-stimulated noncanonical ECs pyroptosis is dependent on the cleavage of caspase-11 and GSDMD, the formation of N-GSDMD pores on the mitochondria, and the release of mtDNA (mitochondrial DNA) (53). The interaction of LPS with HMGB1 would amplify caspase-11-dependent ECs pyroptosis (86). Besides, the upregulated HDAC11 triggers the activity of caspase-3 and GSDME *via* interacting with ERG (ETS-related gene), consequently decreasing ERG acetylation and inducing pyroptosis of TNF-α-treated HUVECs (49).

Accumulating studies have found that the inhibition of ECs pyroptosis can effectively mitigate ECs-mediated inflammation and thrombosis. Melatonin (N-acetyl-5-methoxytryptamine), a neuroendocrine hormone, is characterized with superior antioxidant properties. It has been utilized for forfending vascular ECs pyroptosis by improving mitochondrial function and reducing UQCRC1 (ubiquinol-cytochrome c reductase core protein 1) methylation (87). Interestingly, melatonin can regulate long noncoding RNA MEG3/miR-223/NLRP3 axis to prevent ECs pyroptosis and attenuate the expression of canonical pyroptosis-related molecules (88).

In addition, medicines extracted from natural materials have shown a great anti-pyroptotic value. Neferine is an alkaloid ingredient from the lotus seed embryo, it can reduce ROS generation and block LPS/ATP-induced ECs pyroptosis *via* inactivating ROS/NLRP3/caspase-1 signaling (89). DHM (dihydromyricetin), a natural flavonoid, exerts anti-oxidative and anti-inflammatory bioactivities. DHM markedly abrogates intracellular ROS and mtROS (mitochondrial ROS) generation, further blocking NLRP3-dependent pyroptosis in vascular ECs (90). SAL (salidroside) inhibits the activation of caspase-1, GSDMD, and IL-1β in HUVECs, indicating that SAL impedes aortic ECs pyroptosis (91). Colchicine, a classical nonspecific anti-inflammatory traditional drug, can alleviate cholesterol crystal-induced ECs pyroptosis through activating AMPK/SIRT1 (Sirtuin1) pathway (92).

## 6 The role of pyroptosis in trophoblasts

Trophoblasts, the key cells at the maternal-fetal interface, play an essential role in placenta implantation and reproduction. Pyroptosis of trophoblasts, a unique cell death pathway, occurs in the placenta to aggravate APS-related APOs, as characterized by the elevated proinflammatory cytokines, oxidative stress, and the highly-expressed pyroptosis-related proteins (54) (**Table 1**).

Exposure of trophoblasts to hypoxia elicits excessive UPR (unfolded protein response) activity and ER (endoplasmic reticulum) stress, but also impairs autophagy (54). Hyperactivated UPR upregulates TXNIP (thioredoxin-interacting protein) expression, boosting NLRP3 inflammasome, and subsequently promoting the production of active caspase-1, GSDMD, IL-18 and IL-1β (54). What’s more, attenuated autophagy triggers trophoblasts pyroptosis to expand inflammation and deteriorate APS-related APOs (54). In addition, several biomolecules implicate in modulating trophoblasts pyroptosis. For instance, HOXA9 boosts AMPK/TXNIP/NLRP3 inflammasome by directly increasing chemerin transcription in H/R (hypoxia/reoxygenation)-stressed trophoblasts, further hastening inflammation and pyroptosis of trophoblasts, and aggravating PE. The pathogenic role of chemerin might trigger APS-correlated APOs, providing an effective target for protecting against APOs (55).

Non-coding RNAs (ncRNAs) also exert prominent functions in inducing trophoblasts pyroptosis. A study has detected the increased lncRNA NEAT1 (nuclear paraspeckle assembly transcript 1) expression in LPS-treated trophoblasts, and it might participate in the development of APS-related APOs by inducing pyroptosis. Overexpressed lncRNA NEAT1 heightens TLR4 transcription by competitively binding to miR-302b-3p, and further enhancing LPS-induced trophoblasts pyroptosis together with the increased caspase-1 and N-GSDMD activity (57). MiR-124-3p is also related with trophoblasts pyroptosis, and its expression is increased in the placental tissues of PE patients. The upregulated miR-124-3p triggers pyroptosis by decreasing PLGF (placental growth factor) expression and enhancing ROS production accompanied by the upregulated NLRP3, caspase-1 and GSDMD (56). On the contrary, LINC00240 is lowly-expressed in the placenta tissues of PE patients. The downregulated LINC00240 aggravates oxidative stress-induced pyroptosis through increasing miR-155 and reducing Nrf2 expression, and LINC00240 overexpression mitigates the pyroptotic death of trophoblasts (58). Additionally, the lowly-expressed miR-520c-3p is observed in the placental tissues of PE patients and H/R-treated trophoblasts, and melatonin may suppress trophoblasts pyroptosis *via* modulating miR-520c-3p/SETD7 axis (93). Taken together, these ncRNAs are expected to be possible therapeutic targets for APS-correlated APOs treatment

Recently, trophoblasts pyroptosis has been reported to be modulated by glycolytic enzymes, showing the tightly relation of cellular pyroptosis with metabolic phenotypes. PFKFB3 (6-Phosphofructo-2-kinase/fructose-2,6-bisphosphatase 3) is a widely accepted glycolytic enzyme. Pharmacological MET (metformin) dramatically impedes TLR4/NF-*κ*B/PFKFB3 signaling, which not only rectifies glycometabolic reprogramming and oxidative stress, but also represses NLRP3-induced trophoblasts pyroptosis. Thus, MET-alleviated pyroptosis is partly attributed to PFKFB3-dependent glycometabolism reprogramming and redox disorders, proposing MET as a potential therapeutic approach for APOs (59).

## 7 The role of pyroptosis in platelets

Platelets are small anucleate multifunctional blood cells, implicating in APS-correlated APOs *via* modulating coagulation, thrombosis, inflammation, and innate immunity. Most importantly, inflammasomes activation in platelets is a key participant in upregulating pore-forming proteins and inducing pyroptotic cell death by expressing TLRs (94) (**Table 1**).

Observations have found that platelets pyroptosis is mainly modulated by TLR4/ROS activation. For instance, sepsis-derived S100A8/A9 induces platelets pyroptosis in a GSDMD-dependent manner *via* boosting TLR4/ROS/NLRP3/caspase-1 pathway, leading to the release of oxidized mtDNA and the formation of NETs. NETs in turn release S100A8/A9 and accelerate platelets pyroptosis, forming a positive feedback loop to amplify proinflammatory cytokines production in APS (60). HMGB1 also boosts NLRP3 inflammasome and its adaptor molecule ASC *via* activating TLR4/ROS signaling, resulting in platelets pyroptosis and thrombocytopenia (61, 62). Thus, antioxidant drugs might effectively hinder platelets pyroptosis in APS by reducing ROS secretion. For instance, pretreating platelets with antioxidant NAC (N-acetylcysteine) significantly downregulates the level of NLRP3, cleaved caspase-1, IL-1β, and decreases the proportion of pyroptotic platelets (61, 62). Besides, ND (nanodiamond) has been identified as a carrier for diagnostic and therapeutic platforms. Nevertheless, an investigation has indicated that ND stimulates platelets aggregation and pyroptosis to trigger thrombocytopenia, which is dependent on P-selectin-induced mitochondrial superoxide production and NLRP3 inflammasome activation (63). Thereby, administration of ND with lower doses might reduce platelets-related adverse effects (63).

Of particular, platelets are capable of boosting pyroptosis of macrophages and neutrophils *via* enhancing NLRP3 transcription, ASC oligomerization, caspase-1 activity, and IL-1β secretion. These platelets-mediated effects are independent of cell-to-cell contact, consequently stimulating the expression of calcium-sensing receptors on macrophages (95). Hence, platelets provide an additional regulation for NLRP3 inflammasome and IL-1β-driven pyroptosis (95). On the other side, the increased caspase-11 and NLRP3 in platelets pyroptosis effectively activate the TF secretion from platelets, macrophages, and neutrophils, which facilitates thrombosis in APS development (96).

## 8 The potential of pyroptosis-associated medicines in APS therapy

**Figure 2:**
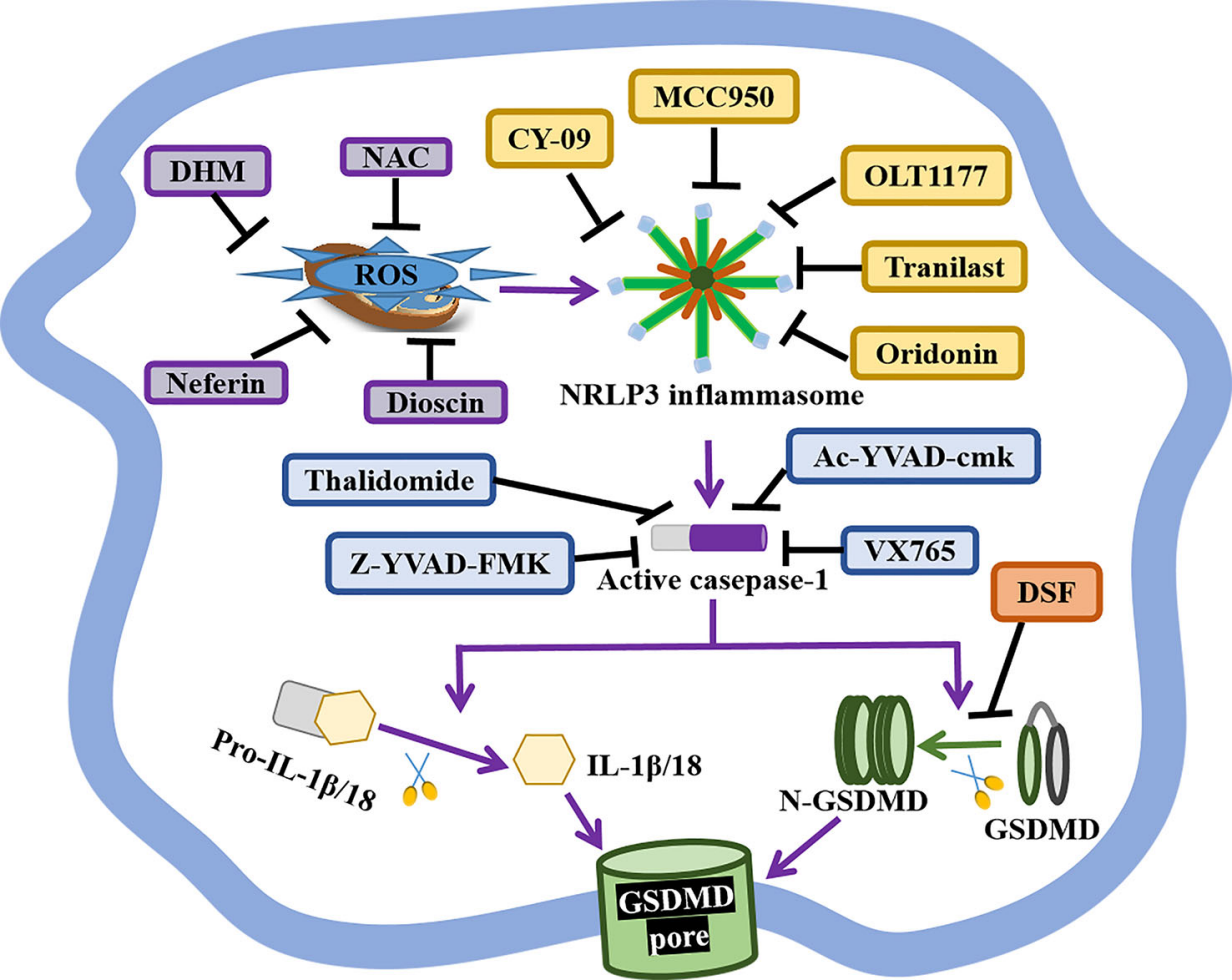
The status of GSDMD, NLRP3 inflammasome, and caspase-1-related inhibitors. GSDMD, NIRP3 inflammasome, and caspase-1 are indispensable in the canonical pyroptosis pathway, and corresponding inhibitors have shown great therapeutical potential for APS. DSF is an inhibitor of GSDMD pores formation. The selective NLRP3 inhibitor MCC950, OLT1177, Tranilast, Oridonin, CY-09 specifically inhibit NLRP3 inflammasome activity and pyroptotic cell death. As well, the anti-oxidant Neferine, DHM, NAC and Dioscin block NLRP3-mediated pyroptosis *via* reducing the production of ROS. Caspase-1 inhibitor Ac-YVAD-cmk, VX765, Thalidomide, Z-YVAD-FMK markedly repress pyroptosis by forfending caspase-1 activation.

Present studies strongly indicate the crucial role of pyroptosis in the pathogenesis and progression of autoimmune diseases, especially canonical pyroptosis. Thus, pyroptosis-based treatment strategies might improve the therapeutic efficacy of APS, such as GSDMD, NLRP3 inflammasome, and caspase-1-related medicines (**Figure 2** and **Table 2**).

**Table 2:** The potential of drugs targeting GSDMD, NLRP3, and caspase-1 in APS therapy.

Drug names	Targets	Therapeutic mechanisms	Refs
DSF	GSDMD	Triggering the covalent modification of GSDMD at Cys191/Cys192 to abrogate pyroptosis, NETs release, and IL-1β secretion	(97, 98)
Neferine	NLRP3	Reducing ROS generation and hindering LPS/ATP-induced pyroptosis *via* blocking ROS/NLRP3/caspase-1 signaling	(89)
DHM	NLRP3	Abrogating ROS generation, and blocking NLRP3-dependent pyroptosis	(90)
NAC	NLRP3	Downregulating the level of NLRP3, cleaved caspase-1, IL-1β, and blocks pyroptosis	(61, 62)
Dioscin	NLRP3	Prohibiting the production of ROS, the expression of NLRP3, caspase-1, and IL-1β	(99)
MCC950	NLRP3	Repressing NLRP3-mediated pyroptosis by hindering ASC/caspase-1/N-GSDMD axis	(100)
OLT1177	NLRP3	Hindering canonical and noncanonical NLRP3 activation, and mitigating caspase-1 and IL-1β activity	(101)
Tranilast	NLRP3	Binding to the NACHT domain of NLRP3 to block the NLRP3–NLRP3 interaction and ASC oligomerization	(102)
Oridonin	NLRP3	Interacting with cysteine 279 of NACHT *via* to prevent the NEK7–NLRP3 interaction and NLRP3 inflammasome activation	(103)
CY-09	NLRP3	Binding with the ATP-binding motif of NLRP3 NACHT domain to forfend NLRP3 ATPase activity and NLRP3 oligomerization	(104)
Ac-YVAD-cmk	Caspase-1	Prohibiting pyroptosis and inflammation *via* blocking IL-6/STAT3/Bcl-6 and caspase-1 activity	(105)
VX765	Caspase-1	Curbing caspase-1/GSDMD pathway and downregulating IL-1β and IL-18	(106)
Z-YVAD-FMK	Caspase-1	Repressing pyroptosis by forfending ROS/NLRP3/caspase-1 activation	(62)
Thalidomide	Caspase-1	Acting as an anti-inflammatory drug by significantly impeding caspase-1 activity	(107)

GSDMD is the crucial effectors of pyroptosis, and the inhibition of GSDMD might prevent inflammation and thrombosis in APS (**Figure 2** and **Table 2**). A study has reported that GSDMD succination blocks its interaction with caspases, attenuating its oligomerization, and limiting its capacity to induce inflammation and pyroptotic cell death (108). DSF, as FDA-approved drug for treating alcohol addiction, is an inhibitor of pores formation by specially triggering the covalent modification of Cys191/Cys192 in GSDMD. DSF abrogates pyroptosis, NETs release, and IL-1β secretion, showing a potential application in APS treatment (97, 98).

NLRP3 inflammasome takes part in diverse hallmarks of APS progression, so NLRP3-based APS treatment is valuable (**Figure 2** and **Table 2**). Dioscin is a new NLRP3 inflammasome inhibitor, it significantly prohibits the production of ROS, the expression of NLRP3, caspase-1, and IL-1β in dioscin-treated macrophages (99). Likewise, the selective NLRP3 inhibitor MCC950 ameliorates inflammation and pyroptosis in macrophages *via* hindering ASC/caspase-1/N-GSDMD axis (100). OLT1177, as an active β-sulfonyl nitrile, it specifically hinders NLRP3 inflammasome activation *in vitro*, but also mitigates caspase-1 activity and IL-1β production in monocytes from CAPS patients and attenuates the severity of LPS-induced systemic inflammation *in vivo* (101). Tranilast, the analog of a tryptophan metabolite, is recognized as an anti-allergic agent. It directly binds to the NACHT domain of NLRP3 to block the NLRP3–NLRP3 interaction and the subsequent ASC oligomerization, showing the great therapeutic and preventive effects on NLRP3-related diseases (102). Oridonin is the major bioactive constituent of Rabdosia Rubescens, it binds to cysteine 279 of NACHT *via* a covalent bond to prevent the NEK7–NLRP3 interaction and the NLRP3 inflammasome activation, but has no effect on AIM2 or NLRC4 activation, LPS-induced NLRP3, IL-1β and, TNF-α production (103). Besides, the ATPase activity of NLRP3 may be a potential drug candidate for the treatment of NLRP3-related diseases, like CY-90. CY-09 can directly bind to the ATP-binding motif of NLRP3 NACHT domain and forfend NLRP3 ATPase activity and NLRP3 oligomerization (104). Moreover, CY-09 is the first well-recognized compound to specifically inhibit NLRP3 inflammasome both *in vitro* and *in vivo*, and its inhibitory mechanism has been clearly elucidated (104).

Caspase-1, as an upstream effector, is also crucial for pyroptosis, and selective caspase-1 inhibitors are worth developing (**Figure 2** and **Table 2**). Caspase-1 inhibitor Ac-YVAD-cmk prohibits humoral immunity response in EAMG (experimental autoimmune myasthenia gravis) rats *via* suppressing IL-6/STAT3/Bcl-6 pathways, providing insights for the development of APS therapy methods (105). Caspase-1 inhibitor VX765 restrains pyroptosis by restraining caspase-1/GSDMD pathway to alleviate colitis in mice, indicating a dose-dependent therapeutic effect on APS (106). Caspase-1 inhibitor Z-YVAD-FMK markedly represses pyroptosis of platelets by forfending ROS/NLRP3/caspase-1 activation (62). Thalidomide is an effective anti-inflammatory drug that significantly impedes caspase-1 activity, it might become a promising agent to better APS therapy (107).

## 9 Conclusion

Evidence of pryroptosis has been gradually detected in APS-correlated cells, which has an impact on the activity of inflammasomes and the generation of prothrombotic and proinflammatory cytokines. Clarifying the related mechanisms might be beneficial for deeply exploring the pathogenesis of APS and developing new therapeutic biomarkers for APS. The selective inhibitors of GSDMD, inflammasomes, and caspase-1 are identified to ameliorate inflammation and thrombosis, and some of them exhibit excellent therapeutic efficacy in several autoimmune diseases. Thereby, these pyroptosis-correlated inhibitors might bring about valuable options for APS therapy. Of particular, the combination of pyroptosis-correlated drugs with common anticoagulant or anti-inflammatory medicines might be more effective for APS treatment, and further studies should focus on verify their clinic safety in APS patients.

## Author contributions

YT designed and wrote the review, drew the figures and tables. QL, ZL and SY wrote and revised this manuscript. LC revised this manuscript and reviewed the figures and tables. All authors contributed to the article and approved the submitted version.

## Glossary

**Table:**

APS	antiphospholipid syndrome
APOs	adverse pregnancy outcomes
CAPS	catastrophic APS
DAMPs	danger-associated molecular patterns
ERG	ETS-related gene
ER	endoplasmic reticulum
ECs	endothelial cells
HUVECs	human umbilical vein endothelial cells
HMGB1	high mobility group box 1
HDAC11	histone deacetylase 11
H/R	hypoxia/reoxygenation
IL	interleukin
LPC	lysophosphatidylcholine
LDH	lactate dehydrogenase
LPS/Ng	LPS plus nigericin
MD-2	myeloid differentiation-2
mtDNA	mitochondrial DNA
mtROS	mitochondrial ROS
NETs	neutrophil extracellular traps
PAPS	primary APS
PTX3	pentaxin 3
PLGF	placental growth factor
PFKFB3	6-Phosphofructo-2-kinase/fructose-2,6-bisphosphatase 3
PAMPs	pathogen-associated molecular patterns
PFD	pore-forming domain
PI	propidium iodide
RIPK3	receptor-interacting serine/threonine protein kinase 3
ROS	reactive oxygen species
RCN2	reticulocalbin-2
RD	repressor domain
RA	rheumatoid arthritis
RAGE	TLR4/advanced glycation end product
SDHB	succinate dehydrogenase complex subunit B
SIRS	systemic inflammatory response syndrome
TF	tissue factor
TRAF3	tumor necrosis factor receptor-associated factor 3
TXNIP	thioredoxin-interacting protein
TNF	tumor necrosis factor
TLRs	toll-like receptors
ULK1	unc-51 like autophagy activating kinase 1
UQCRC1	ubiquinol-cytochrome c reductase core protein 1
UPR	unfolded protein response.
